# Novel bioprocessing strategies and evaluation models for walnut meal bioactives

**DOI:** 10.1016/j.fochx.2026.103670

**Published:** 2026-02-20

**Authors:** Fengling Tan, Yachun Chen, Bing Qi, Siting Li, Zhou Chen, Aijin Ma, Yingmin Jia

**Affiliations:** a School of Food and Health, Beijing Technology and Business University, Beijing 100048, China; b Hebei Key Laboratory of Walnut Nutritional Function and Processing Technology, Hengshui 053000, China

**Keywords:** Walnut meal, Bioactive components, Solid state fermentation, Organoid model

## Abstract

This review addresses the challenges in valorizing walnut meal, a by-product of walnut oil extraction, by outlining its key bioactive constituents—proteins/active peptides, polysaccharides, and phenolic compounds—and their health benefits. It examines emerging green processing strategies, including mixed-strain solid-state fermentation, microbe-enzyme synergistic fermentation, and integrated multi-technology approaches. The article also discusses the evolution of efficacy assessment from conventional cellular and animal models toward human biomimetic platforms such as organoids and organs-on-chips, which offer more physiologically relevant models for elucidating functional mechanisms. In contrast to existing reviews focusing on isolated components or conventional techniques, this work constructs an integrated framework spanning from “green synergistic processing” to “human biomimetic evaluation.” It aims to accelerate the translation of walnut-derived bioactive compounds into functional foods and pharmaceuticals, promoting comprehensive, high-value utilization of walnut resources.

## Highlights

• Novel processing strategies enhance yield and sustainability of bioactives.
• Mixed-strain SSF and enzyme synergy enhances component bioavailability.
• Organoid models enable precise human-relevant bioactivity evaluation.
• Multi-organ chips holistically assess systemic health benefits of bioactives.

## 1 Introduction

Globally recognized as a healthful lipid source, walnuts are primarily processed to extract their nutritious oil. However, the substantial by-product generated after oil extraction—walnut meal—remains underutilized, often discarded or relegated to low-value feed applications, thereby representing a substantial loss of resources (Lakhlifi El Idrissi et al., 2024). The presence of impurities such as walnut shell fragments, septa, and seed coats in the meal hinders its further processing. Currently, most walnut meal is discarded or used as low-value feed, leading to resource waste and environmental concerns. Therefore, developing advanced technologies for its high-value utilization is urgently needed. Recent studies have revealed that walnut meal is rich in various bioactive components, including proteins, bioactive peptides, polysaccharides, and phenolic compounds, indicating significant nutritional and health potential. However, conventional extraction and efficacy evaluation methods severely limit its in-depth exploitation. To address these challenges, this review systematically summarizes the main bioactive components and health benefits of walnut meal, with a focus on novel processing strategies such as mixed-strain solid-state fermentation (SSF), microbial-enzyme synergistic catalysis, and multi-technology integration, which significantly enhance extraction efficiency and sustainability. Additionally, it examines the evolution of evaluation models from traditional cell/animal assays to organoid systems, highlighting organ-on-a-chip technology as a next-generation research tool. This advanced approach holds great potential for simulating human metabolic environments and elucidating the health effects and mechanisms of walnut meal bioactive compounds, thereby providing new directions for the resource utilization and health product development of walnut meal.

## 2 Bioactivity and functional properties of walnut meal

**Fig. 1:**
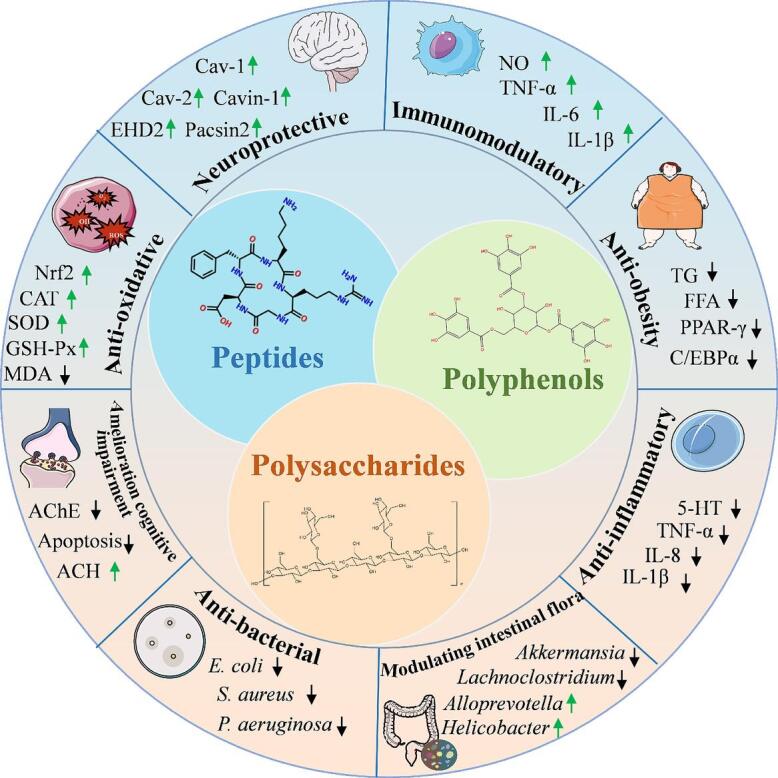
Functional active components in walnut meal and their health benefits.

As a major by-product of walnut oil extraction, walnut meal is enriched with a diverse profile of essential nutrients beyond lipids. Recent studies have revealed that it contains various nutritional and pharmacologically active components, which confer high nutritional value and broad health benefits as shown in Fig. 1. These compounds contribute to reducing free radical levels, improving cognitive function, and play a positive role in mitigating the risk of cardiovascular diseases, depression, dementia, and type 2 diabetes mellitus (Ni et al., 2022).

### 2.1 Walnut protein and bioactive peptides

Walnut meal is a plant protein resource with high nutritional value. Its protein content is as high as 40%, including 18 amino acids (including 8 essential amino acids). According to solubility, it can be divided into albumin, globulin, prolamin and glutelin. However, due to poor solubility, this high-quality protein resource has not been effectively utilized for a long time. In recent years, with the increasingly prominent role of food-borne functional factors in the adjuvant treatment of diseases, bioactive peptides (composed of 2–20 amino acid residues) extracted from walnut proteins by enzymatic hydrolysis, fermentation or chemical synthesis have received extensive attention (Wen et al., 2020). Compared with intact proteins, these small molecular peptides not only have better bioavailability and can be absorbed and utilized by the human body more efficiently, but also play a key role in a variety of cellular functions and physiological regulation. Studies have shown that bioactive peptides derived from walnut meal have rich physiological activity and medicinal value. Compared with traditional therapeutic drugs, these bioactive components exhibit advantages such as lower toxicity and fewer side effects, demonstrating significant potential in improving human health and disease prevention, thereby making the efficient utilization of walnut protein highly valuable for increasing by-product added value and achieving comprehensive resource utilization. Walnut protein is a high-quality plant protein. A large number of studies have reported that the enzymatic hydrolysate of walnut protein after enzymatic treatment also shows a variety of biological activities as shown in Table 1. Recent studies have shown that walnut peptides prepared by complex enzymatic hydrolysis technology not only have good flavor characteristics, but also exhibit better antioxidant activity (Shen, Fang, et al., 2024). Especially for walnut peptides with a molecular weight of less than 3 kDa, their digestion products significantly enhance antioxidant capacity due to structural characteristics (tertiary structure weakening, secondary structure random coiling). Among them, KGHLFPN peptide was proved to have effective DPP-IV inhibitory activity (Li, Li, et al., 2023). Low molecular weight peptide tripeptide leucine-proline-phenylalanine (LPF) can effectively alleviate the symptoms of colitis by reducing apoptosis, anti-inflammatory effects and regulating intestinal flora (Zhi et al., 2022). WSREEQEREE and ADIYTEEAGR improve UV-induced light by inhibiting the NF-κB/MMP-1 signaling pathway in female rats (Xu et al., 2020). In terms of nervous system protection, walnut peptide TWLPLPR (TW-7) maintains the integrity of the blood-brain barrier by inhibiting the expression and activity of matrix metalloproteinase 9, which can improve the learning and memory ability of mice (Dang et al., 2022). Similarly, the octapeptide WCPFSRSF has been shown to improve memory function (Zhang, Zheng, et al., 2023). The current research is also committed to optimizing the sensory characteristics of walnut peptides. By reducing the production of bitter peptides and developing bitter masking techniques, its application potential in functional foods has been significantly improved (Zhang, Bai, et al., 2023). These systematic findings provide a solid scientific basis for the development of targeted functional foods based on walnut peptides.

**Table 1:** Preparation and functional properties of major bioactive components from walnut.

Walnut bioactive components	Walnut origin	Functional activity	Treatment methods	Results	Reference
Walnut protein and active peptide	Walnut variety “Xin 2” from Chacha Food Co., Ltd., Hefei, China	Anti-inflammatory activity	*In vitro* gastrointestinal digestion simulation	Three walnut peptides with potent anti-inflammatory properties were found: IPAGTPVYLINR, FQGQLPR, and VVYVLR.	(Xia et al., 2024)
	Walnut-derived peptide synthesized by Jiangsu Ji Tai Peptide (Yancheng, China)	Neuroprotective effect	Artificial synthesis	The walnut-derived peptide EVSGPGYSPN (EV-10) showed significant neuroprotective effects *in vivo*.	(Li et al., 2024)
	Peptides WCPFSRSF synthesized by GL Biochem Ltd. (Shanghai, China).	Memory improving effect	Artificial synthesis	Octapeptide WCPFSRSF improved the memory of mice.	(Zhang, Zheng, et al., 2023)
	Persian walnut, the Chandler variety	Anticancer properties	Protease hydrolysis	The peptide fraction of walnut meal protein hydrolysate inhibited the growth of breast cancer and colon cancer cells by 63 ± 1.73% and 51 ± 1.45%, respectively.	(Jahanbani et al., 2016)
	Walnuts (*Juglans regia L*.) were obtained from Aksu Industrial Base (Xinjiang, China) in 2022	Angiotensin-converting enzyme inhibition	Ultrasound-assisted extraction	Two novel angiotensin-converting enzyme (ACE) inhibitory peptides, tyrosine-isoleucine-glutamine (YIQ) and isoleucine-tyrosine-glutamine (IYQ).	(Wang, Tang, et al., 2023)
	Walnut-derived peptide synthesized by Jiangsu Ji Tai Peptide (Yancheng, China)	Alleviation of oxidative stress in colitis	Artificial synthesis	The walnut-derived peptide LPLLR (LP-5) can effectively improve the oxidative stress of DSS-induced colitis by activating the NRF2 pathway.	(Qi, Wu, et al., 2023)
	Walnut-derived peptide synthesized by Jiangsu Ji Tai Peptide (Yancheng, China)	Improve learning ability and cognitive disorders	Artificial synthesis	Ameliorates hippocampal neuronal damage and preserves blood-brain barrier (BBB) integrity.	(Qi, Wang, et al., 2023)
	Synthesized by Shanghai Sangon Bioengineering Technology Service Co., Ltd. (Shanghai, China)	Improvement of DSS-induced ulcerative colitis injury	Synthetic peptide	Three novel walnut protein peptides (SHTLP, HYNLN and LGTYP) were found to improve intestinal mucosal barrier dysfunction and reduce inflammation by inhibiting the activation of TLR4-MAPK pathway *in vivo*.	(Hong et al., 2023)
	Walnut-derived peptide synthesized by Nanjing Jetide (Nanjing, Jiangsu, China)	Alleviate memory impairment caused by sleep deprivation	Artificial synthesis	The walnut-derived peptide WCPFSRSF alleviates the damage to the hippocampus of mice and inhibits oxidative stress by regulating antioxidant enzymes and NRF2 pathways.	(Zhang et al., 2022)
	Walnut-derived peptide synthesized by Jiangsu Ji Tai Peptide (Yancheng, China)	Antioxidant and neuroprotective activities	Artificial synthesis	Mitochondria mediated by putative kinase 1 (PINK1) regulated by C-Jun N-terminal kinase (JNK) improved oxidative stress-mediated neuronal damage.	(Yang et al., 2022)
	Coldly squeezed degreased walnut meals Shangluo City, southeast Shanxi province, China	Reduce serum uric acid levels in hyperuricemia rats	Alcalase 2.4 L protease	Walnut meal hydrolysate (WMH) and dephenolized walnut meal hydrolysate (DWMH) can effectively reduce serum uric acid levels and protect renal function in potassium oxonate-induced hyperuricemia rats *in vivo*.	(Li et al., 2018)
	Walnut meal, Yunnan Moore Farm Food Technology Co., Ltd.	Antiobesity effect	Alkaline protease, pH 9.0, 3 h	Enzymatic hydrolysate of walnut meal can inhibit lipid accumulation in 3 T3-L1 adipocytes, and its mechanism is related to the inhibition of lipid synthesis-related gene expression.	(Bai, Peng, et al., 2024)
Walnut polyphenols	Walnut from Hebei Golden Dragon Co. Ltd. (Handan, China)	Antibacterial activity	Microbiological fermentation	Among the 15 phenolic compounds of fermented walnut meal, salicylic acid (SA) showed the strongest antibacterial activity against *Penicillium* in *Rosa roxburghii*.	(Hu et al., 2024)
	Walnuts in Romania	Anti-inflammatory activity	Walnut septum was mixed with water/acetone (50:50, *v*/v) at a ratio of 1:10 (*w*/*v*)	The content of α-tocopherol in walnut septum extract was significantly higher than that of γ-and δ-tocopherol, and the expression of inflammatory factors (IL-6, IL-8, IL-1β) in HGF cells was decreased.	(Rusu et al., 2020)
	Xinjiang thin-skinned walnut was purchased from Jinpincheng Food Store, China	Inhibition of acetylcholinesterase (AChE) activity.	Walnut kernels without seed coat and defatted walnut meal.	Walnut seed coat polyphenols can induce changes in the secondary structure and amino acid composition of walnut protein, and enhance the inhibitory activity of acetylcholinesterase (AChE).	(Su et al., 2024)
	Walnut germplasm resource nursery in Shanxi	Antioxidant activity	Desiccation treatment at 40 °C	The contents of protocatechuic acid and 4-hydroxybenzoic acid in walnut seed coat were the highest, showing significant antioxidant effect.	(Li, Wang, et al., 2023)
	Lvling Co., Ltd. (Hebei Province, China), and all of the walnuts were harvested in October 2021	Antidepressant activity	Ethanol, ethyl acetate extraction	Pretreatment with walnut polyphenols (75 and 150 μg/mL) could significantly reverse the decrease of cell viability.	(Zou et al., 2023)
	Lvling Co., Ltd. (Hebei Province, China), and all of the walnuts were harvested in October 2021	Neuroprotective effect	Ethanol, ethyl acetate extraction	Walnut protein and its active metabolite urolithin A (UroA) can exert neuroprotective effects against oxidative damage by up-regulating the PKA/CREB/BDNF signaling pathway in SH-SY5Y cells treated with hydrogen peroxide.	(An et al., 2023)
	The walnut meals were from Huizhiyuan Food Ltd., Yunnan, China.	Inhibition of 3 T3-L1 preadipocyte differentiation	Extracted with 50% ethanol and purified by HPD-100 macroporous resin.	Effectively improve the symptoms of metabolic syndrome, inhibit weight gain, regulate glucose and lipid metabolism disorders, but also alleviate liver injury.	(Liang et al., 2017)
Walnut polysaccharide	Walnuts (Juglans sigillata Dode) from a local market in Yunnan, China	Treat constipation	Compound enzymatic hydrolysis	Walnut insoluble dietary fiber reduces functional constipation by regulating the intestinal motility axis of intestinal microorganisms.	(Yang et al., 2024)
	Chilean in-shell walnuts obtained Bengaluru, India	Antibacterial activity	Microwave-assisted solvent extraction	Inhibitory effects on Gram-negative bacteria (*Pseudomonas aeruginosa* MTCC 424) and positive bacteria (*Staphylococcus aureus* MTCC96).	(Sindhu et al., 2024)
	Walnut distraction wood: Dali Yangbi Walnut Co., Ltd.	Anti-inflammatory activity	Water extraction and ethanol extraction	The aqueous extract and alcohol extract of *Juglans regia* can inhibit the secretion and expression of inflammatory factors and MAPK signaling pathway induced by lipopolysaccharide in RAW264.7 cells to reduce the inflammatory response.	(Gao et al., 2024)
	Walnut green husk Bozhou Hengyi Traditional Chinese Medicine Technology Co., Ltd	Improve liver inflammation	Water extraction and alcohol precipitation.	Walnut green husk polysaccharide improves ochratoxin A-induced liver inflammation and gluconeogenesis disorders in mice by regulating intestinal flora	(Yang et al., 2023)
	an agricultural prod- uct trading market in Xi'an, Shaanxi Province	immunoregulation	Solvent extraction method (immunoregulation 95% ethanol and water extraction method combined)	WGHP-2 activates Raw264.7 macrophages by regulating MAPK and PI3K/Akt signaling pathways: not only enhances cell phagocytic activity, but also promotes cell proliferation.	(Wang, Yan, et al., 2023)
	Fresh walnut green husk was obtained from a local market (Xi'an, Province, China)	Prevention of chronic diseases induced by high fructose diet	Solvent extraction method (95% ethanol and water extraction method combined)	Protective effects of walnut green husk polysaccharide on liver injury, vascular endothelial dysfunction and intestinal flora disorder induced by high fructose in mice.	(Wang et al., 2020)
	Walnut green husks were obtained from a local farmers market (Xi'an, China)	Protecting the integrity of intestinal barrier function	Solvent extraction method (95% ethanol and water extraction method combined)	*Juglans regia* green husk polysaccharide can prevent obesity, chronic inflammatory response, nonalcoholic fatty liver disease and colon tissue damage in high-fat diet	(Wang, Yang, et al., 2021)
	Walnut fruits with green hulls in the south of Italy,	–	Alkaline and acidic extracts	The differences in producing areas significantly affected the content, morphological characteristics and thermal stability of the extracted glucan and pectin.	(La Torre et al., 2021)
	Green walnut husks were purchased from the local market (Shangluo, China)	Antioxidant and anticancer activities	Extraction method using 85% methanol solution	The heteropolysaccharides isolated and purified from the exocarp of *Juglans regia* showed strong anticancer activity by inhibiting cell proliferation and increasing intracellular lactate dehydrogenase level.	(He et al., 2020)
	Walnut diaphragm from Kunming city, China	Anti-tumor and immune-enhancing effects	Microwave-assisted extraction	Walnut diaphragm polysaccharides effectively inhibited the proliferation of HepG2 and BGC-823 tumor cells and activated RAW264.7 macrophages through CR3/MR/TLR2 receptors.	(Meng et al., 2018)

The efficient preparation and application of walnut bioactive peptides currently face multiple challenges and opportunities. To obtain high-yield and high-purity active peptides, it is necessary to optimize the enzymatic preparation process and develop a controllable delivery system (Wen et al., 2023). The large-scale application of walnut bioactive peptides is still limited by insufficient separation and purification technologies. While the low solid-to-liquid ratio enzymatic hydrolysis process used in the laboratory stage can improve protein conversion rates, it leads to excessive water consumption and a sharp increase in subsequent concentration and drying costs during industrial-scale production (Liu et al., 2022). In the future, the development of green and efficient production processes, such as the use of ultrasound, high pressure and other physical field assisted enzymatic hydrolysis technology, combined with gradient infiltration enzyme membrane reactor and intelligent monitoring methods, can significantly improve the efficiency of enzymatic hydrolysis. In-depth analysis of the structure-activity relationship of bioactive peptides to elucidate the mechanisms by which novel processing technologies improve the functional properties of walnut protein. In addition, the use of biological simulation, peptide structure-activity analysis and mathematical modeling techniques will help to reduce R & D costs (Wen et al., 2023). These systematic studies will provide important theoretical support for the functional food development and precise nutrition utilization of walnut protein and its active peptides.

### 2.2 Walnut phenolic compounds

*Juglans regia*, the tree species richest in polyphenols among woody nuts, contains a variety of phenolic compounds in its seed coat and distraction wood, with free phenolics dominating throughout the developmental stages and being significantly more abundant in the seed coat than that in the kernel (Cui et al., 2024). Walnut is the most polyphenol-rich species among woody nuts (Persic et al., 2018), with its seed coat, septum (internal woody tissue), and other tissues all containing abundant phenolic compounds. Studies have found that free phenolic compounds predominate throughout all developmental stages, with their content in the seed coat being significantly higher than that in the kernel. Studies have shown that its seed coat is rich in phenolic substances such as hexahydroxybiphenyldicarboxylic acid-digalloylhexose isomer and vanillic acid hexoside, which makes its antioxidant activity significantly better than other nuts (Persic et al., 2018). The phenolic compounds in walnut kernel and seed coat were characterized by ultra-high performance liquid chromatography-tandem mass spectrometry. A total of 40 compounds were identified, including 13 phenolic acids (non-flavonoids), 12 flavonoids, 8 flavanols, 4 alkaloids, 1 tannin, 1 proanthocyanidin and 1 naphthoquinone (Shen et al., 2021). The contents of protocatechuic acid and 4-hydroxybenzoic acid in walnut seed coat were the highest (>400 μg/g), and the three phenolic forms had significant antioxidant activity (Li, Wang, et al., 2023).

Walnut polyphenols exhibit extensive biological activities as shown in Table 1, daily intake of phenolic-rich foods or beverages can reduce the risk of all-cause death and chronic diseases (Parmenter et al., 2025). In terms of neuroprotection, extracts can reduce the risk of Alzheimer ‘s disease by reducing Aβ-mediated cytotoxicity and reducing neuronal loss (Muthaiyah et al., 2011). The active metabolite of walnut protein, urolithin A, can exert neuroprotective effects against oxidative damage by up-regulating the PKA/CREB/BDNF signaling pathway in SH-SY5Y cells treated with hydrogen peroxide (An et al., 2023). Concerning anti-tumor, polyphenols and ellagic acid can inhibit the proliferation of tumor cells (Anderson & Teuber, 2010). *Juglans regia* L. significantly inhibited the viability, proliferation and migration of human A172 glioblastoma cells. In addition, most ciprofloxacin-resistant Gram-positive and Gram-negative bacteria have significant antibacterial effects (Genovese et al., 2020). Regarding metabolic regulation, 12 polyphenols were identified in the cold-pressed defatted walnut meal extract. Among them, walnut A, gallic acid, ellagic acid and ellagic acid-4-O-xyloside were the main components, which could improve glucose and lipid metabolism disorders and enhance liver antioxidant activity (Liang et al., 2017). Notably, polyphenols not only enhance the acetylcholinesterase (AChE) inhibitory activity by altering the secondary structure of walnut proteins (Su et al., 2024), but also significantly improve the oxidative stability of walnut oil (Emre, 2019). As the main by-product of oil extraction, walnut meal is rich in both proteins and polyphenolic compounds. Studies have found that bioactive peptides released from walnut meal through complex enzymatic hydrolysis exhibit a synergistic effect with coexisting polyphenols, significantly enhancing AChE inhibitory activity. Based on these findings, this study utilizes walnut meal as the raw material and employs controlled enzymatic hydrolysis to deeply process its protein components, aiming to develop functional peptide-based food ingredients with memory-improving properties. This strategy not only provides an effective approach for the value-added utilization of walnut processing by-products but also provides novel insights for functional food development.

### 2.3 Walnut polysaccharides

Natural polysaccharides have the advantages of rich sources, high safety, less adverse reactions, and significant biological activity, and are expected to become potential candidate natural compounds for clinical functional supplements or drug development (Teng et al., 2025). Walnut and its processing by-products are rich in a variety of polysaccharides with significant biological activities, as shown in Table 1, showing broad development prospects.

Studies have shown that walnut green husk polysaccharide (WGHP) is a low molecular weight acidic heteropolysaccharide, mainly composed of rhamnose, galactose and galacturonic acid (Wang et al., 2020). The polysaccharide has multiple physiological functions: in terms of metabolic regulation, it can prevent liver damage and oxidative stress induced by high fructose or high fat diet by regulating intestinal flora (Wang, Yang, et al., 2021); in terms of immune regulation, WGHP isolated from green walnut peel can significantly promote the proliferation of RAW264.7 cells, enhance phagocytic activity, and up-regulate the production of various inflammatory factors (Wang, Yan, et al., 2023). In addition, walnut insoluble dietary fiber (WIDF) is rich in galacturonic acid and glucose, which can alleviate constipation by activating serotoninergic synapses and inhibiting opioid receptor/inducible nitric oxide synthase (Oprd/iNOS) signaling pathway (Yang et al., 2024). The water-soluble polysaccharide fraction (DJP-2) isolated from walnut distraction wood is mainly composed of glucose, galactose, arabinose, xylose and trace mannose, showing significant antioxidant and antibacterial activities (Meng et al., 2017). These natural polysaccharides have the advantages of abundant sources, high safety, and significant biological activity (Teng et al., 2025). WGHP have been shown to have significant anti-liver injury, vascular injury, intestinal barrier injury, intestinal flora regulation, immune regulation and prevention of obesity, and have no cytotoxicity (Wang et al., 2020; Wang, Yang, et al., 2021). WGHP has a protective effect on liver steatosis and vascular endothelial dysfunction. It can reverse the intestinal flora imbalance caused by high fructose diet (Wang et al., 2020), and improve ochratoxin A-induced liver inflammation and gluconeogenesis disorders in mice by regulating intestinal flora (Yang et al., 2023). With the in-depth study of the structure-activity relationship of walnut polysaccharides (Zhou & Huang, 2023), these natural products are expected to become important functional supplements for the prevention and treatment of chronic diseases.

## 3 Novel processing methods for the extraction of functional active components from walnut meal

**Fig. 2:**
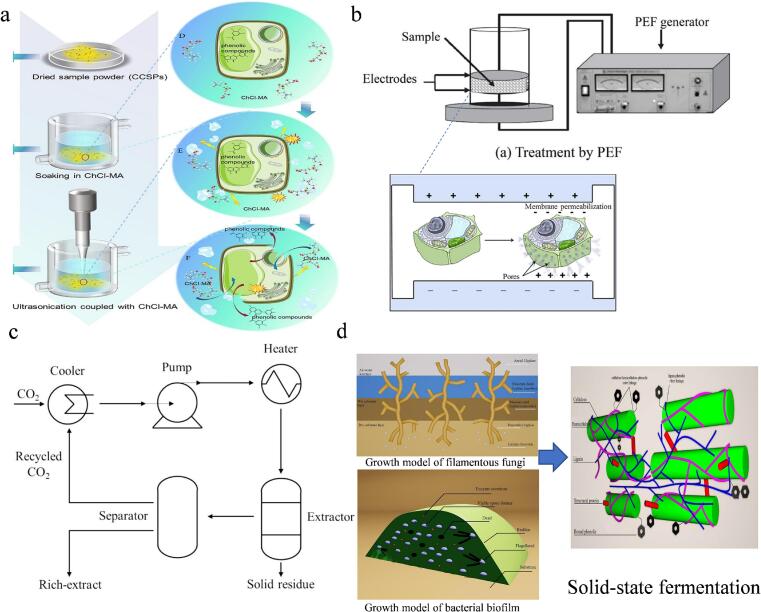
Novel processing methods for the extraction of functional bioactive compounds from walnut meal. (a) Ultrasound-assisted natural deep eutectic solvent (Fu et al., 2021); (b) pulsed electric field-assisted extraction (Xi et al., 2021)); (c) supercritical carbon dioxide fluid extraction (Ma et al., 2025); (d) mixed-strain SSF (De Villa et al., 2023).

The traditional methods for preparing active components (such as proteins/peptides, phenols, and polysaccharides) in walnut meal mainly include organic solvent extraction (such as ethanol extraction of phenols), alkali-soluble acid precipitation (extraction of proteins), and hot water extraction (extraction of polysaccharides). However, these methods suffer from significant limitations, including the health hazards of residual organic solvents (*e.g.*, n-hexane), the degradation of heat-sensitive components (such as oxidation of polyphenols and denaturation of proteins) under high temperatures or extreme pH, low extraction efficiency (*e.g.*, insufficient polysaccharide yield), and cumbersome multi-step purification processes. These drawbacks make the synergistic extraction of multiple components challenging, resulting in resource waste and reduced bioactivity (Xi et al., 2021). In contrast, modern green extraction techniques such as electron beam radiation (Shen, Shi, et al., 2024), pulsed electric field (Xi et al., 2021) as shown in Fig. 2. Environmentally friendly methods such as natural deep eutectic solvents (NADES) show significant advantages, which can not only reduce solvent residues, but also improve the extraction efficiency of active ingredients. For example, the excellent extraction effect of NADES on polyphenols from grape pomace provides a basis for its application in food by-products. At the same time, strategies such as mixed-strain SSF, microbe-enzyme synergistic fermentation, and multi-method synergistic extraction are shown in Table 2, which provides a more sustainable solution for the efficient preparation of active ingredients from walnut meal.

**Table 2:** Novel processing method for the key bioactive components release.

Treatment	Functional active factor	Key conclusions	Modification effects	Reference
Sonication-synergistic natural deep eutectic solvent	Phenolic compounds from peels of *Carya* *cathayensis* Sarg	Natural deep eutectic solvents (NADES, choline chloride-malic acid system, ChCl-MA) and ChCl-MA pulsed ultrasound combined technology have significant synergistic effects in the yield of active ingredients and antioxidant activity.	This method achieves a higher yield than conventional approaches, utilizing biodegradable solvents and energy-efficient ultrasonication in a cost-effective process, though scale-up requires further validation.	(Fu et al., 2021)
*L. plantarum* B7 and *B. subtilis* fermentation	Walnut peptides and phenolics	When walnut pentapeptide YVVPW (1.0 mg/mL) was combined with salicylic acid (SA) (0.27 mg/mL), the synergistic effect was the most significant, showing the strongest antibacterial activity.	Microbial fermentation transformation offers an environmentally friendly and cost-effective approach, though its scalability requires further validation.	(Hu et al., 2024)
Ultrasonic assisted cellulase hydrolysis	Polyphenols from Walnut Septum	Walnut distraction wood polyphenols have strong antioxidant activity.	As a green technology, ultrasound-assisted enzymatic extraction demonstrates effective extraction with low energy consumption, high efficiency, and favorable scalability.	(Bai, Zhang, et al., 2024)
Electron beam radiation processing	Walnut protein and its hydrolysates	The changes of secondary structure, tertiary structure and surface morphology of walnut meal protein (WMP) and walnut meal protein hydrolysate (WMPHS) caused by EBI treatment improved their functional properties.	An energy-efficient non-thermal processing technology, effectively enhances the functional properties of plant proteins while operating without extensive chemical solvents.	(Shen et al., 2024)
Mixed SSF by *A. niger* and *R. oryzae*	Pomegranate pomace polyphenol	A 5-day fermentation resulted in a peak bound polyphenol content of 26.50 mg GAE/g DW, representing a 3.22-fold increase over the unfermented control.	Mixed-culture SSF significantly enhances polyphenol yield through a solvent-free, low-cost process with favorable scalability.	(Gao et al., 2025)
NADES combined with ultrasonic and microwave irradiation assisted technology.	Grape residue phenols	NADES has good extraction effect on polyphenols (especially polyphenols in grape pomace), and has good antioxidant and antiproliferative activity of tumor cell lines.	This method achieves a notably higher extraction yield than conventional ethanol extraction, utilizing a cost-effective solvent with distinct environmental advantages.	(Panic et al., 2019)
Acidic NADES system of high-speed homogenization-cavitation blasting extraction (HSH-CBE) technology	Fresh mulberry anthocyanins	The total extraction amount of anthocyanin was 6.05 mg/g fresh weight, which was 1.24 times higher than that of traditional organic solvent method.	This extraction method demonstrates superior efficiency compared to traditional organic solvent-based techniques, albeit requiring more complex procedures and advanced equipment.	(Guo et al., 2019)
**Supercritical carbon dioxide fluid extraction technology using ethanol as modifier**	Antioxidant activity	The total phenol content in the exocarp of walnut was significantly increased.	This technology employs fully recyclable green solvents throughout the process, thereby avoiding the environmental contamination caused by conventional solvents, albeit requiring substantial equipment investment.	(Wenzel et al., 2017)
*Bacillus subtilis* and alkaline protease synergistic solid-state fermentation	Antimicrobial peptides	The antibacterial peptide content of walnut glutelin reached 26.35 ± 1.29 mg/g; it has obvious antibacterial effect on *Staphylococcus aureus*.	This modification method exhibits superior efficacy, low energy consumption, and notable environmental benefits.	(Ma et al., 2024)
Mixed-strains SSF	Nattokinase	The double strains combined with SSF of cold-pressed walnut meal improved the activity of nattokinase, significantly reduced the ammonia flavor of the product, and improved the flavor of traditional fermented products.	This fermentation method exhibits markedly improved efficiency and demonstrates favorable environmental benefits, though its cost-effectiveness requires further validation through scale-up production.	(Wang et al., 2017)

### 3.1 Mixed-strain SSF

The growth and product formation of microorganisms on solid particles under the condition of no free water (or near absence) in SSF technology can realize the high-value utilization of by-products of agricultural products processing, which has attracted wide attention (Wang, Li, et al., 2024). Due to its cost-effectiveness and environmental advantages, SSF is an important means in the preparation of active components of walnut meal. The single strain treatment often has the defect of low fermentation efficiency, and the mixed strain culture can help to improve the SSF effect by the metabolic synergy between the strains (Shahab et al., 2020). From the perspective of microbial interaction mechanisms, the intrinsic rationale behind enhanced bioactive compound release efficiency in mixed-strain SSF can be analyzed as follows: different microbial strains engage in metabolic division of labor, secreting complementary enzyme systems that synergistically degrade plant matrices, thereby rupturing cell walls and facilitating product synthesis. Quorum sensing fine-tunes microbial behavior in a density-dependent manner, enhancing community adaptation to low-water-activity environments. Spatial heterogeneity is exploited through niche differentiation, where aerobic and facultative anaerobic bacteria cooperate to optimize conditions within fermentation microniches. Cross-conversion of by-products reduces undesirable off-odors while simultaneously generating flavor compounds, achieving a dual enhancement in functionality and flavor profile. The cold-pressed walnut meal by *Bacillus natto* and *Saccharomycopsis Dalekai* combined with SSF improved the nattokinase activity of the product. The nattokinase activity of the fermented walnut meal reached 2040.82 U/g, an increase of 70.42%; the ammonia flavor of mixed strains fermented products was significantly reduced, which improved the flavor of traditional fermented products (Wang et al., 2017). Similarly, using walnut meal as raw material, the fermentation performance of walnut meal polypeptide prepared by SSF of *Bacillus subtilis* 10,160 and *Aspergillus niger* GIM3.452 was compared and investigated (Liu et al., 2018). In the future, rational design of mixed microbial systems through synthetic biology approaches—such as editing microbial secretory enzyme profiles, quorum-sensing pathways, and substrate utilization preferences—can further enhance the function-oriented performance of microbial communities in SSF, enabling efficient and targeted release of bioactive compounds. Leveraging the structural and compositional characteristics of walnut meal, synthetic biology and genome-editing approaches will be employed to optimize SSF systems using microbial consortia, thereby enabling efficient release of functional bioactive components.

### 3.2 Microbe-enzyme synergistic fermentation

The synergistic fermentation technology combines the advantages of microbial fermentation and enzymatic hydrolysis technology. Enzymatic hydrolysis can provide basic nutrients such as nitrogen source and carbon source for the fermentation process of microorganisms. At the same time, fermentation technology can also overcome the lack of functional elements caused by simple enzymatic hydrolysis. Antimicrobial peptides derived from walnut glutelin were prepared *via* a three-day SSF using *Bacillus subtilis* in combination with alkaline protease (400 U/g). The resulting product exhibited a peptide content of 26.35 ± 1.29 mg/g and demonstrated significant inhibitory activity against *Staphylococcus aureus*. This microbe-enzyme synergistic SSF approach not only overcomes the limitations associated with single enzymatic hydrolysis or microbial fermentation alone, but also improves the stability of antimicrobial peptides, which provides a new research idea for the high-value utilization of walnut meal (Ma et al., 2024). The underlying mechanism of this synergy involves dynamic interactions where microbial metabolism modulates the fermentation microenvironment (*e.g.*, pH, ionic strength), thereby optimizing enzymatic activity and stability. Concurrently, enzymatic hydrolysis breaks down complex proteins into readily assimilable peptides and amino acids, which not only support microbial growth but also stimulate the expression of microbial proteases and functional metabolites. This reciprocal relationship enhances the selective release and retention of bioactive peptides while reducing inhibitory by-products, thereby improving the yield and stability of target compounds such as antimicrobial peptides. Using the enzyme production and enzymatic hydrolysis ability of microorganisms, the protein is hydrolyzed into small molecular peptides and amino acids. Protein hydrolysates not only contribute to improving food flavor but have also been confirmed to possess certain functional activities. Therefore, microbial-enzymatic synergistic fermentation represents a pivotal approach for valorizing walnut meal, as it enhances nutritional properties by elevating both the content and bioavailability of functional bioactive compounds.

### 3.3 Synergistic multi-modal extraction method

Multi-physical field synergistic strategy has shown significant advantages in the efficient green extraction of active components from walnut meal (Yang et al., 2025). Compared with the traditional organic solvent extraction method (with the risk of solvent residue, the destruction of heat-sensitive components and high energy consumption), the enzymatic hydrolysis combined with shear emulsification method can make the dietary fiber of defatted walnut meal form a loose and porous honeycomb structure, and improve the viscosity and emulsifying activity (Khan et al., 2018). Pulsed electric field technology achieves high yield at room temperature through non-thermal effect and reduces the amount of solvent, but the electric field strength needs to be optimized to avoid the degradation of active ingredients caused by hydroxyl radicals (Song et al., 2025; Xi et al., 2021). In addition, ultrasound-microwave assisted NADES can efficiently extract polyphenols due to its green and safe characteristics (Fu et al., 2021; Panic et al., 2019). Electron beam irradiation coupled with ultrasound-assisted NADES extraction significantly enhanced the yield of proanthocyanidins from walnut husks, reaching 56.34 mg/g, a 32.93% increase over conventional methods. The extracted proanthocyanidins demonstrated promising antioxidant activity and digestive enzyme inhibition capacity (Qi et al., 2025). For polysaccharides, microwave-assisted extraction combined with response surface optimization of walnut diaphragm polysaccharide extraction process can not only improve the yield, but also retain its immunomodulatory and anti-tumor activity (such as activating macrophage CR3/MR/TLR2 receptor pathway) (Meng et al., 2018). Supercritical CO_2_ fluid extraction technology (with ethanol as modifier) further broke through the limitations of traditional methods, and showed higher total phenol content in the extraction of polyphenols from black walnut peel (Wenzel et al., 2017). The intrinsic mechanism of multi-technology synergy lies in the targeted and complementary disruption of the plant matrix. Different physical fields (*e.g.*, electric, ultrasonic, microwave) and biological agents (*e.g.*, enzymes) act on distinct structural levels—cell wall, membrane, and molecular bonds. This cascade effect sequentially breaks down structural barriers, enhances mass transfer, and facilitates the release of encapsulated compounds while minimizing degradation, as the milder conditions of one method can offset the intensiveness of another. These multi-technology synergy strategies can improve the extraction efficiency and reduce the environmental burden, while retaining the structural and functional integrity of the active ingredients to the greatest extent, providing a new direction for the high-value utilization of walnut meal.

As summarized, the novel processing strategies—mixed-strain SSF, microbe-enzyme synergistic fermentation, and multi-mode cooperative extraction—each possess distinct characteristics. Mixed-strain SSF offers notable cost-effectiveness and environmental advantages, with no solvent residues and the ability to optimize product flavor, making it a vital approach for high-value utilization of walnut meal. However, it currently faces challenges such as relatively low yield of active components, insufficient stability of the fermentation system, and limited scalability due to uneven fermentation in solid substrates. Microbe-enzyme synergistic fermentation integrates the strengths of microbial fermentation and enzymatic hydrolysis, outperforming single methods in terms of active component yield and bioavailability, and effectively compensating for the shortcomings of either process alone. Nevertheless, it requires precise control of reaction conditions to ensure efficacy, leading to higher production costs and restricted scalability for large-scale industrial applications. Multi-mode cooperative extraction achieves the highest yield, relying on green technologies for efficient extraction while maximally preserving the structural and functional integrity of active components with minimal environmental impact. Its limitations, however, include high equipment investment and operational costs, and the fact that some technical combinations lack mature systems for scaled application. Looking forward, SSF will continue to play a key role. By integrating synthetic biology and genome editing tools, the fermentation system can be redesigned to overcome bottlenecks such as inadequate retention of activity and low yield. Multi-method cooperative strategies should be further advanced, incorporating green physical field pretreatment combined with targeted enzymatic hydrolysis and microbe-enzyme synergistic solid-state fermentation. This integrated physico-biological approach will enhance both extraction efficiency and bioactive preservation, ultimately contributing to the development of an intelligent, green, and synergistic extraction system.

## 4 Model systems for assessing walnut-derived bioactives

**Fig. 3:**
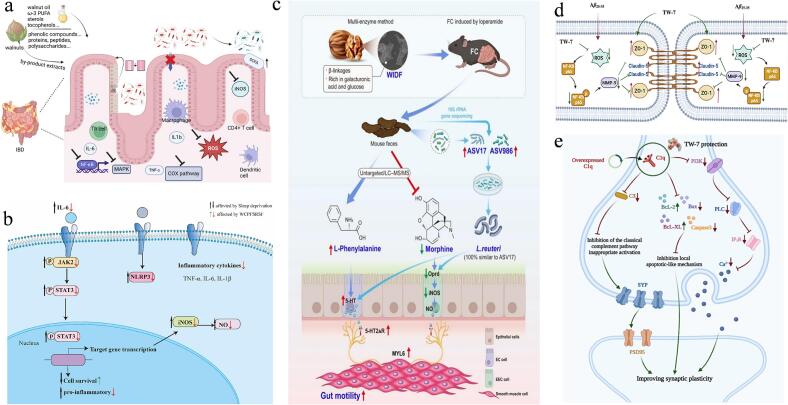
Health effects and mechanisms of bioactive factors from walnut meal ((a) regulatory effects of walnut-derived bioactive components on intestinal inflammation (Dai et al., 2024); (b) mechanism of walnut peptides in inhibiting neuroinflammation in sleep-deprived mice (Zhang et al., 2022); (c) mechanism of walnut insoluble dietary fiber in alleviating constipation (Yang et al., 2024); (d) mechanism of walnut peptides in protecting blood-brain barrier integrity and improving memory (Dang et al., 2022); (e) schematic diagram of the mechanism by which walnut peptides protect HT22 cells against C1q-related neuroinjury (Xing et al., 2023).)

Walnut meal is rich in a variety of active ingredients such as proteins, polysaccharides and polyphenols. A variety of models are used to comprehensively evaluate its functional characteristics. This evaluation strategy based on component-function-application can provide accurate scientific basis for the high-value utilization of walnut meal as shown in Fig. 3.

### 4.1 Cell model

The cell model study systematically revealed the multiple biological effects of walnut active ingredients as shown in Table 3. In terms of anti-tumor, walnut protein hydrolysate showed 63 ± 1.73% and 51 ± 1.45% growth inhibition rates on MDA-MB231 and HT-29 cancer cells, respectively (Jahanbani et al., 2016). The walnut diaphragm extract (high polyphenol content) inhibited the proliferation of A549, T47D-KBluc and MCF-7 cancer cells by selective cytotoxicity (Rusu et al., 2020). Neuroprotective studies have shown that walnut polyphenols can alleviate nerve injury by up-regulating the PKA-CREB-BDNF pathway in PC12 cells (Zou et al., 2023), which can effectively inhibit the cytotoxicity induced by β-amyloid and improve Alzheimer ‘s disease (Muthaiyah et al., 2011). Concerning antioxidant, walnut protein hydrolysate had a protective effect on H_2_O_2_-induced oxidative damage in PC-12 cells (Wang, Liu, et al., 2022). Walnut peptide LPLLR (LP-5) significantly reduced ROS levels and enhanced SOD and CAT activities in Caco-2 cells (Qi, Wu, et al., 2023). Anti-tumor studies have shown that walnut diaphragm extract can inhibit the proliferation and migration of A172 glioblastoma cells (Genovese et al., 2020). The exocarp polysaccharide of green walnut enhanced the anticancer activity by increasing lactate dehydrogenase release (He et al., 2020). In terms of metabolic regulation, cold-pressed defatted walnut meal extract significantly inhibited the differentiation of 3 T3-L1 preadipocytes (Liang et al., 2017), which is expected to become a potential clinical candidate for the prevention and treatment of metabolic syndrome. In addition, the nano‑silver composite material prepared by walnut green husk polysaccharide WGHP2 (WGHP2-Ag) has significant antibacterial activity and has no obvious toxic effect on PC12 cells (Wang, Yang, et al., 2024), while walnut distractor extract (DJF-W/DJF-E) exerts anti-inflammatory effects on lipopolysaccharide-induced RAW264.7 cells through flavonoids and polyphenols (Gao et al., 2024). These studies have confirmed the multiple biological activities of walnut active ingredients in anti-oxidation, anti-tumor, metabolic regulation and anti-inflammatory. While cell models provide fundamental mechanistic insights at the molecular level, they lack the systemic complexity of a whole organism. To bridge this gap, several *in vivo* and more complex *in vitro* models are employed.

**Table 3:** Application of different models in the evaluation of walnut active ingredients.

Model types	Research object	Active ingredient	Results	Reference
Nematode model	*C. elegans*	Walnut diaphragm antioxidant constituents	Prolonged lifespan and reduced the accumulation of lipomycin in the *C. elegans* model.	(Hong et al., 2021)
Drosophila model	*Drosophila melanogaster*	walnut oil	Walnut oil significantly improved the exercise capacity of rotenone-exposed fruit flies and reduced their mortality.	(Avallone et al., 2024)
	*Drosophila melanogaster*	walnut peptide	Tetrapeptide LPQF showed significant improvement in type 2 diabetes.	(Chen et al., 2025)
Zebrafish model	Scopolamine-Induced Zebrafish	Walnut protein source arginine peptide	Improved scopolamine-induced cognitive memory impairment in zebrafish.	(Wang, Su, et al., 2022)
	Zebrafish exposed to bisphenol AF	walnut peptide	Significantly improve the cognitive and memory dysfunction of zebrafish.	(Wei et al., 2024)
	Zebrafishes (wild type AB strain, 6 months old)	Walnut protein hydrolysate	Walnut protein hydrolysates have significant neuroprotective activity.	(Wang et al., 2021)
	Scopolamine-induced zebrafish	Walnut peptide LR and LPI	LR and LPI improve learning and memory function by regulating cholinergic system, synaptic developmental plasticity, neurotrophic factors and oxidative stress.	(Wang et al., 2025)
Cellular model	LPS-treated NCM460 cells	walnut polypeptide	Polypeptides can effectively reduce the expression levels of 5-hydroxytryptamine (5-HT), tumor necrosis factor-α (TNF-α) and vascular endothelial growth factor (VEGF), and reduce apoptosis and inflammatory response.	(Xia et al., 2024)
	H_2_O_2_ induced HT22 cell model.	Walnut-derived peptides TWLPLPR(TW-7)	Down-regulation of C1q expression, thereby reducing the level of complement C3, while increasing the expression of presynaptic membrane protein SYP and postsynaptic density protein PSD95.	(Xing et al., 2023)
	Human breast cancer (MDA-MB231) and colon cancer (HT-29) cell lines.	Walnut (*Juglans regia* L.) Protein Hydrolysates	The antioxidant activity of walnut protein hydrolysate was directly correlated with its anticancer activity, indicating that it has potential therapeutic application value.	(Jahanbani et al., 2016)
	Human neuroblastoma (SH-SY5Y)	walnut polyphenols	Walnut polyphenols and its active metabolite urolithin A improved oxidative damage in SH-SY5Y cells by up-regulating the PKA / CREB / BDNF signaling pathway.	(An et al., 2023)
	Human A172 glioblastoma cells	Walnut diaphragm extract	*Juglans regia* L., an extract from the diaphragm of *Juglans regia* L., inhibits the dual effects of A172 glioblastoma cell survival and bacterial growth.	(Genovese et al., 2020)
	Human gingival fibroblasts HGF	High phenolic content walnut diaphragm extract	Reduce the expression of inflammatory factors (IL-6, IL-8, IL-1β) in HGF cells, reflecting the anti-inflammatory effect.	(Rusu et al., 2020)
	RAW264.7 cell	Walnut green husk polysaccharide	Significantly promotes the proliferation of RAW264.7 cells and enhances their phagocytic activity.”	(Wang, Yan, et al., 2023)
	LoVo cells	Polysaccharides from green walnut husk	Significantly inhibits tumor cell proliferation and demonstrates stronger anti-cancer activity	(He et al., 2020)
	HepG2 and BGC-823 tumor cells	Walnut diaphragm polysaccharide	Walnut diaphragm polysaccharides effectively inhibited the proliferation of HepG2 and BGC-823 tumor cells and activated RAW264.7 macrophages through CR3/MR/TLR2 receptors.	(Meng et al., 2018)
Mouse model	Gender disturbance, female rats	Walnut peptide	Walnut peptides WSREEQEREE and ADIYTEEAGR inhibit NF-κB/MMP-1 signaling pathway in female rats.	(Xu et al., 2020)
	Male 57BL/6J mice	Walnut-derived peptides EVSGPGYSPN	Caveolin CAV-1 promotes targeted delivery of walnut-derived peptides to the brain.	(Li et al., 2024)
	Type 2 diabetes mellitus rats	Walnut powder phenolic substances	Exhibits anti-diabetic effects by modulating glucose and lipid metabolism in T2DM rats.	(Li et al., 2020)
	Loperamide-induced constipation mouse	Water-insoluble dietary fiber rich in galacturonic acid and glucose	Effectively alleviates loperamide-induced constipation in mice.	(Yang et al., 2024)
	Ochratoxin A damaged mice	Walnut green husk polysaccharide	Exerts significant protective effects against ochratoxin A (OTA)-induced hepatic inflammation and gluconeogenic dysfunction in mice.	(Yang et al., 2023)
Organoids model	Human brain organs	Theanine (Th) and walnut peptide	Downregulates serotonin transporter expression and upregulates brain-derived neurotrophic factor.	(Zhong et al., 2025)

### 4.2 Zebrafish model

Walnut protein and its hydrolysate showed significant neuroprotective effects in zebrafish model, which could improve cognitive dysfunction and anxiety behavior induced by various factors. Studies have shown that walnut peptides (such as components prepared by alkaline protease hydrolysis) can significantly up-regulate the expression of neurotrophic factors BDNF and GDNF in zebrafish brain, and improve cognitive and memory impairment caused by bisphenol AF (1 μg/mL) exposure. The mechanism may be related to promoting the expression of neurotrophic factors and alleviating oxidative stress (Wei et al., 2024). In addition, walnut protein-derived arginine peptides showed neuroprotective effects in scopolamine-induced zebrafish cognitive impairment model, mainly by regulating cholinergic neurotransmitter levels and enhancing antioxidant enzyme activity (Wang, Su, et al., 2022). Further studies have found that walnut protein hydrolysate can repair the cholinergic system damaged by scopolamine and alleviate oxidative stress, thereby improving memory function (Wang, Su, et al., 2021). In the anxiety model, walnut peptides (such as Val-Tyr, VY) significantly reduced PTZ-induced excessive locomotor behavior in zebrafish larvae, showing an anti-anxiety effect. In addition, it is predicted that peptides LR and LPI can not only transport across Caco-2 cell monolayer in a complete form (LR actively transports and bypasses passive diffusion through PepT1, LPI depends on PepT1), but also improve learning and memory function through multiple pathways, including regulation of cholinergic system, synaptic plasticity, neurotrophic factors and oxidative stress (Wang et al., 2025). These studies have shown that the neuroprotective effect of walnut peptides in zebrafish model involves a variety of molecular mechanisms, which provides a scientific basis for its application in cognitive and emotional disorders intervention. Complementing vertebrate models like zebrafish, invertebrate models offer unique advantages for high-throughput genetic screening and lifespan studies, particularly in metabolic and aging research.

### 4.3 Invertebrate models

*Drosophila melanogaster,* as an ideal model for the study of metabolic diseases, has shown important value in revealing the neuroprotective and metabolic regulation effects of walnut and its active ingredients. In the study of neurodegenerative diseases, after rotenone (Rot) induced Parkinson ‘s disease phenotype in *Drosophila melanogaster,* walnut oil supplementation significantly improves motor dysfunction and reduce mortality. The protective mechanism is closely related to the antagonism of polyunsaturated fatty acids in walnut oil on rotenone neurotoxicity (Avallone et al., 2024). In the study of metabolic disorders, the active peptides obtained from the hydrolysis of walnut protein by bromelain (such as tetrapeptide LPQF) showed significant anti-diabetic potential: In the high-sugar diet (HSD) -induced insulin resistance Drosophila model, LPQF effectively improved T2DM -like symptoms by inhibiting DPP-IV activity and regulating Wnt, MAPK and FoxO signaling pathways (Chen et al., 2025). These studies not only confirmed the applicability of the Drosophila model in the study of metabolic disease mechanisms, but also revealed the multi-target mechanism of walnut-derived bioactive substances in neuroprotection and blood glucose regulation. In addition, *Caenorhabditis elegans* is a classic model organism. The walnut diaphragm extract CDG prolongs the lifespan of *C. elegans*, increases resistance to heat and oxidative stress, and reduces the accumulation of lipomycin in the *C. elegans* model (Hong et al., 2021). Despite their utility for studying specific phenotypes and pathways, invertebrate and lower vertebrate models have inherent physiological differences from mammals. Mammalian animal models remain indispensable for evaluating systemic effects, organ interactions, and complex behaviors relevant to human health.

### 4.4 Animal model

The animal model plays a key role in the functional evaluation of the active components of walnut meal. Studies have shown that the walnut peptide WCPFSRSF peptide alleviates nerve damage in sleep-deprived mice through the NRF2 pathway (Zhang et al., 2022); the walnut peptide LPLLR can improve the learning and memory ability of mice with cognitive impairment and alleviate the symptoms of colitis (Qi, Wang, et al., 2023). The protective effect of walnut-derived LPF on colitis in mice and the effect of promoting recovery (Zhi et al., 2022). Walnut protein hydrolysate can reduce alcohol-induced oxidative stress in rat hippocampus (Xu & Zhao, 2022). In terms of metabolic diseases, walnut powder rich in polyphenol extracts showed anti-diabetic effects, which can regulate glucose and lipid metabolism and help to identify potential therapeutic targets for the prevention and intervention of type 2 diabetes (T2DM) (Li et al., 2020). Walnut meal hydrolysate and dephenolized walnut meal hydrolysate can reduce serum uric acid levels in hyperuricemic rats (Li et al., 2018). WGHP improves ochratoxin A-induced liver inflammation and gluconeogenesis disorders in mice by regulating intestinal flora (Yang et al., 2023). Similarly, WGHP effectively improved high-fat-induced intestinal flora disease in rats, and produced short fatty acids by fermentation in the colon to reduce inflammatory damage in the colon and protect the integrity of intestinal barrier function (Wang, Yang, et al., 2021). However, current animal models present notable limitations: physiological and metabolic differences between rodents and humans may compromise the extrapolation of research findings; single-disease models cannot fully reflect the multi-target effects of bioactive components; and most studies are constrained by small sample sizes and insufficient long-term safety evaluations. Furthermore, existing research predominantly focuses on the specific effects of isolated compounds rather than systematically evaluating the synergistic interactions among multiple components in walnut meal, significantly limiting the clinical applicability of these findings. Future studies should prioritize establishing more human-relevant disease models and conducting systematic evaluations incorporating multi-component and multi-target approaches. The limitations inherent in animal models, particularly species differences and ethical concerns, have driven the development of more human-relevant, complex *in vitro* systems. To directly address the need for models that better recapitulate human tissue architecture and function, novel organoid technologies have emerged as a transformative approach.

### 4.5 Emerging organoid models

Traditional models for assessing food-derived bioactive factors, including those described above, face significant limitations when predicting human responses. Two-dimensional cell cultures lack three-dimensional microenvironments and cell-cell interactions, animal models are constrained by species differences, and simpler biological models cannot accurately replicate complex human physiological mechanisms. In recent years, organoids have emerged as groundbreaking three-dimensional culture systems to overcome these gaps. These “mini-organs” derived from human cells can closely mimic the genetic and epigenetic characteristics of target tissues, demonstrating unique advantages in studying dietary bioactive compounds (Glorevski et al., 2016; Tan et al., 2024). In walnut bioactive research, most existing studies rely on conventional *in vitro* cell lines and animal experiments. For example, oxidative stress model of PC12 cells and zebrafish experiments showed that WP (222 μg/mL) could inhibit H_2_O_2_-induced cell death (neuroprotective rate of 42%), and regulate caspase 3/7/8 activity and Bax/BDNF mRNA expression (Liu et al., 2019). However, such models cannot fully simulate the complex neurotransmitter regulatory networks of the human brain, highlighting a gap in clinically translatable research (Liu et al., 2022). The application of human brain organoid models is addressing this need. Pioneering studies using a brain organoid stress (BO-stress) model found that WP combined with theanine (Th) could regulate levels of key neurotransmitters (GABA, 5-HT, DA) and significantly modulate the expression of related transporters and neurotrophic factors (Zhong et al., 2025). Subsequent validation in mouse models confirmed behavioral and cognitive improvements, demonstrating a valuable correlation between organoid predictions and *in vivo* outcomes. Beyond neuroscience, intestinal organoid studies have demonstrated nutrient absorption modulation by bioactive compounds like epicatechin gallate, and colorectal cancer organoid research has shown multi-pathway modulation by curcumin (Chen et al., 2022; Inoue et al., 2022; Yin et al., 2019). Currently, a variety of organoids including small intestine, brain, liver, pancreatic islet, and kidney have been successfully constructed (Tan et al., 2024), providing versatile platforms for walnut bioactivity assessment. Nevertheless, current organoid systems still face challenges such as the absence of vascularization and limited interactions with immune cells. Recent advances in microfluidic technology and 3D bioprinting have substantially improved organoid maturation and microenvironment simulation. Looking ahead, the integration of organoid-on-chip platforms with bioprinting technologies will facilitate the establishment of more robust evaluation systems for walnut bioactive components, thereby accelerating their clinical translation and practical applications.

## 5 Concluding remarks and future perspectives

Efficient extraction and precise efficacy evaluation of bioactive components from walnut meal are crucial for achieving its high-value utilization. In terms of enhancing the bioaccessibility of active compounds, establishing intelligent systems characterized by green and synergistic processes represents a key future trend. SSF will continue to play a vital role in releasing bioactive components from walnut meal due to its cost-effectiveness and environmental advantages. Notably, the integration of mixed-culture SSF with synthetic biology and genome editing tools holds promise for rationally reconstructing fermentation systems, enabling the establishment of stable, efficient, and self-regulating co-culture systems. This approach can overcome current bottlenecks of insufficient activity retention and low yield in practical production. Multi-method synergistic strategies are particularly important; for instance, employing green and energy-efficient physical field pretreatment to disrupt the structure of walnut meal, followed by targeted enzymatic hydrolysis using rationally designed composite enzyme preparations or solid-state fermentation integrating microbes and enzymes. Such integrated physico-biological strategies can simultaneously enhance extraction efficiency and component activity.

At the level of efficacy evaluation, the limitations of traditional animal models are driving the evolution of assessment systems toward greater human relevance and precision. The integration of novel organoid models with organ-on-a-chip technology provides a powerful tool for analyzing the digestion, absorption, metabolic pathways, and health effects of bioactive components. Tao et al. successfully constructed an interactive liver-islet organoid system derived from human induced pluripotent stem cell (hiPSC) using microfluidic technology, which precisely simulates the dynamic response of the liver-islet axis in glucose metabolism regulation on-chip, providing an innovative platform for metabolic disease research (Tao et al., 2022). Intestinal organoids faithfully recapitulate the digestive absorption processes of walnut polyphenols and their interaction mechanisms with gut microbiota, whereas hepatic organoids precisely delineate their metabolic conversion pathways. Together, these complementary models provide critical insights for elucidating the bioavailability and ultimate bioactive forms of these compounds. Regarding the brain-health-specific benefits of walnuts, brain organoid models overcome the limitations of traditional two-dimensional cultures by reconstructing neural circuits within a three-dimensional structure, creating an ideal environment for investigating the regulatory mechanisms of walnut peptides on neurogenesis, synaptic plasticity, and neurotransmitter networks. Furthermore, integrated “multi-organ chip” systems incorporating gut, liver, and brain units can holistically simulate the entire journey of walnut bioactive components—from absorption and metabolism to transport and targeted effects—within the human body, enabling a systematic assessment of their health benefits. Looking ahead, the deep integration of food science, engineering, and data science to establish a complete technological chain from intelligent preparation to systemic evaluation will accelerate the transformation of walnut bioactive components into high-value health products.

## CRediT authorship contribution statement

**Fengling Tan:** Writing – original draft, Investigation, Conceptualization. **Yachun Chen:** Methodology, Investigation. **Bing Qi:** Investigation. **Siting Li:** Supervision, Investigation. **Zhou Chen:** Writing – review & editing. **Aijin Ma:** Validation, Investigation, Funding acquisition. **Yingmin Jia:** Writing – review & editing, Project administration, Funding acquisition, Conceptualization.

## Declaration of competing interest

The authors declare that they have no known competing financial interests or personal relationships that could have appeared to influence the work reported in this paper.

## Data Availability

Data will be made available on request.

## Appendix A Supplementary data

Supplementary material Permissions for Figures. Supplementary material
