# Supplementary material for: Novel bioprocessing strategies and evaluation models for walnut meal bioactives
Source: Food Chem X. 2026 Feb 20;34:103670. doi: 10.1016/j.fochx.2026.103670 (PMC12938866; doi:10.1016/j.fochx.2026.103670)
Supplement: Supplementary material — Permissions for Figures. [file mmc1.docx]

**Titie:** Novel bioprocessing strategies and evaluation models for walnut meal bioactives

**Dear Editor,**

**The permissions for all figures have been attached**

Figure 2 (a) (b) (c) (d)

Figure 3 (a) (b) (c) (d) (e);

**Thanks.**

Figure 2a

Figure 2 b

Figure 2 c

Figure 2 d

Figure 3 a

Figure 3 b

Figure 3 c

Figure 3 d

Figure 3 e
